# AMPA Receptor Trafficking in Homeostatic Synaptic Plasticity: Functional Molecules and Signaling Cascades

**DOI:** 10.1155/2012/825364

**Published:** 2012-05-13

**Authors:** Guan Wang, James Gilbert, Heng-Ye Man

**Affiliations:** Department of Biology, Boston University, 5 Cummington Street, Boston, MA 02215, USA

## Abstract

Homeostatic synaptic plasticity is a negative-feedback response employed to compensate for functional disturbances in the nervous system. Typically, synaptic activity is strengthened when neuronal firing is chronically suppressed or weakened when neuronal activity is chronically elevated. At both the whole cell and entire network levels, activity manipulation leads to a global up- or downscaling of the transmission efficacy of all synapses. However, the homeostatic response can also be induced locally at subcellular regions or individual synapses. Homeostatic synaptic scaling is expressed mainly via the regulation of **α**-amino-3-hydroxy-5-methyl-4-isoxazolepropionic acid receptor (AMPAR) trafficking and synaptic expression. Here we review the recently identified functional molecules and signaling pathways that are involved in homeostatic plasticity, especially the homeostatic regulation of AMPAR localization at excitatory synapses.

## 1. Introduction

The brain has the amazing ability to adapt through its capability to change in response to experience and use. This fundamental property of plasticity serves to learn and remember complex tasks, obtain rewards, or even recover after injury. Surprisingly, with constant dynamic changes occurring in the brain, neuronal activity remains stable over an entire lifespan. Our brains appear to be constructed in such a manner that the mechanisms involved in learning and memory can be balanced by another distinct form of neuronal modulation, homeostatic plasticity. These two forms of plasticity coexist to adapt to the changing sensory world while maintaining a balance of neural activity within a physiological range.

 Hebbian synaptic plasticity is associative and input specific, which strengthens or weakens the transmission efficacy of individual synapses. Long-term potentiation (LTP) and depression (LTD), the two best studied forms of Hebbian plasticity, are widely considered to be the cellular mechanisms for learning and memory. However, given the positive-feedback nature of Hebbian plasticity, this form of synaptic modulation could potentially result in synapses of either functional saturation or silence, driving the whole network into an unstable state if left unchecked. Hebbian synaptic plasticity therefore necessitates distinct homeostatic mechanisms that can stabilize a network in the face of constant dynamic changes in synaptic strength. Indeed, neuronal networks use an array of homeostatic negative-feedback mechanisms that allow neurons to assess their activity and adjust accordingly so as to restrain their activity within a physiological range [1–3].

## 2. Expression of Homeostatic Regulation via Scaling Synaptic Strength and AMPAR Abundance

**Figure 1:**
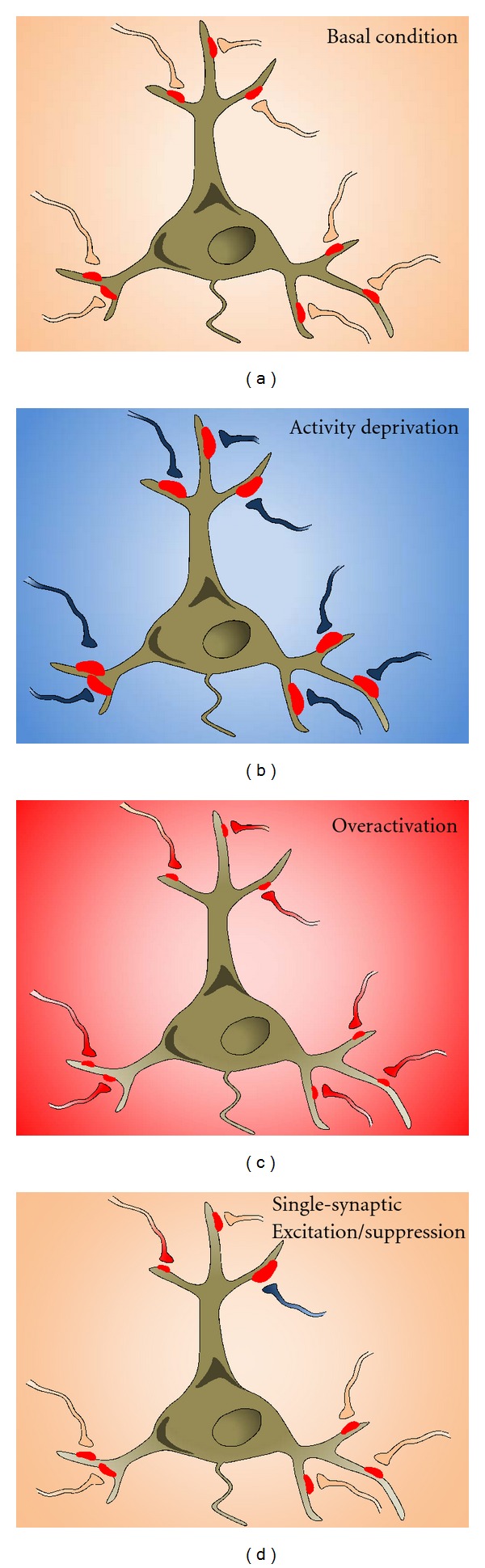
Global and local homeostatic synaptic plasticity. Under basal condition, neurons have a relative stable level of activity as a result of basal synaptic inputs mediated by AMPARs (red clusters) at the postsynaptic domains (a). When the activity of a neuron or a network is chronically suppressed such as by TTX incubation, the strength of all synapses is elevated proportionally via an increase in AMPAR abundance (b). In contrast, an overall reduction in synaptic AMPAR accumulation is induced by lasting overactivation of neuronal activity (c). At individual synapses, long-term synaptic inactivity (terminal in blue) leads to a homeostatic increase in AMPAR amount, whereas lasting synaptic excitation causes selective AMPAR reduction at the stimulated synapse (terminal in red), without affecting the neighboring normal synapses (d).

**Figure 2:**
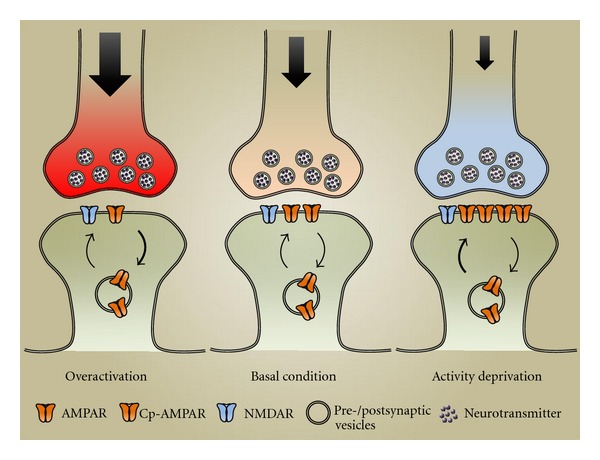
Activity-dependent homeostatic regulation of AMPAR synaptic localization. Under basal conditions, a stable level of AMPARs at postsynaptic surface is maintained by balanced trafficking processes of receptor insertion and internalization (middle). High synaptic activity can be detected by neurons, leading to enhanced receptor internalization and reduced surface receptor localization (left). Conversely, when neurons are treated by long-term activity deprivation, higher levels of AMPARs are expressed at postsynaptic surface via enhanced receptor insertion, including both GluA2-containing and GluA2-lacking receptors (Cp-AMPAR) (right). During homeostatic regulation, the number of NMDARs at postsynaptic surface is not significantly altered under either condition.

At the neuronal level, homeostatic plasticity aims to maintain a stable firing rate of action potentials. This can be achieved through adjustments in the strength of synaptic inputs, neuronal excitability, neuronal connectivity, or the balance between excitation and inhibition. Among these possibilities, regulation of synaptic strength has been the most extensively studied and is believed to be the most crucial measure in homeostatic regulation. This form of regulation, known as homeostatic synaptic plasticity or synaptic scaling [4, 5], is expressed mainly by an alteration in AMPAR synaptic accumulation [1, 6–9]. During homeostatic regulation, AMPAR numbers at the postsynaptic surface are accordingly scaled up- or downwardly in response to activity deprivation or overexcitation, respectively, presumably via changing AMPAR trafficking processes including receptor insertion and internalization (Figures 1 and 2).

In central neurons, the most studied model of homeostatic synaptic plasticity is activity deprivation by a sodium channel blocker, tetrodotoxin (TTX). When cultured cortical neurons are incubated with TTX to chronically abolish action potentials and thus silence network activity, the synapse responds in a compensatory manner, resulting in an increase in the strength of synaptic transmission [4, 10, 11]. By measuring AMPAR-mediated miniature excitatory postsynaptic currents (mEPSCs), it has been shown that chronic suppression of network activity results in an upscaling of synaptic activity [4, 12, 13]. Conversely, chronic network hyperactivation, commonly induced by bath application of bicuculline, an antagonist of the inhibitory gamma-aminobutyric acid A (GABA_A_) receptors, results in a homeostatic downscaling in mEPSCs [4].

## 3. Global and Local Synaptic Homeostatic Plasticity

Homeostatic plasticity has traditionally been considered a response that globally affects the entire synapse population of a neural network or a whole neuron, in which AMPAR accumulation at every synapse is up- or downwardly scaled according to activity manipulation [6, 14]. In practice, global homeostatic regulation is usually induced by bath application of reagents that affect the activity of the whole network, in which mEPSC amplitude and AMPAR synaptic amounts at each cell in the network are affected in a compensatory manner [4]. In addition to a network scale, global homeostatic regulation has also been studied at a level of individual neurons. By overexpression of inward-rectifier potassium channels, Kir 2.1, Burrone et al. show that single neuron inhibition results in a homeostatic upregulation in mEPSCs [15]. Ibata et al. show that inhibition of single neuron activity by local perfusion of TTX at the somatic area induces homeostatic regulation in mEPSC and AMPAR synaptic expression [16]. In a recent study, individual neurons expressing light-sensitive channels are selectively activated for 24 hrs [17]. Using this paradigm, Goold and Nicoll demonstrate that chronic activation of single neurons lead to a homeostatic reduction in AMPAR-mediated synaptic transmission [17].

In contrast to global regulation, recent studies indicate that homeostatic synaptic responses could also occur locally at subcellular regions [8, 14, 17, 18]. In cultured hippocampal neurons, synapses in a small region of dendrites are suppressed by microperfusion of TTX together with the N-methyl-D-aspartate (NMDA) receptor antagonist, APV. Using this method, Sutton et al. demonstrate that rapid synaptic scaling of AMPARs is induced locally at the silenced region [19]. However, in another study, local application of TTX on dendrites fails to alter synaptic AMPAR expression [16]. This discrepancy may be due to the difference between activity manipulation paradigms (with or without NMDA receptor inhibition).

Although findings indicate that the traditionally considered global homeostatic plasticity can be induced at local dendritic areas, whether it occurs at the level of individual synapses, an important question regarding synapse functional stability, has not been studied until recently. Our work shows for the first time that the homeostatic response is indeed employed locally at the single synapse level [18, 20]. In cultured hippocampal neurons, overexpression of Kir2.1 inward-rectifier potassium channels to selectively inhibit the activity of individual presynaptic terminals results in a homeostatic increase in corresponding postsynaptic AMPAR expression [20]. To study the opposite paradigm, Hou et al. selectively activate single synapses by employing a light-gated glutamate receptor [18, 21]. We demonstrate that the level of AMPARs at excited synapses is selectively downregulated via receptor internalization and proteasomal degradation [18]. These findings suggest the existence and autonomous execution of homeostatic mechanisms at individual synapses. Although AMPAR trafficking is likely shared by global and single synaptic homeostatic responses, it remains unclear whether similar or distinct signaling cascades and molecular components are adopted in global versus single-synaptic homeostatic regulation.

## 4. Signaling Molecules and Pathways Regulating AMPAR Trafficking in Homeostatic Plasticity

**Figure 3:**
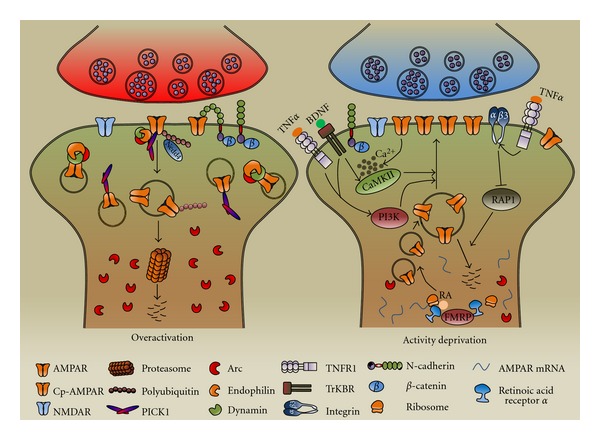
Regulation of homeostatic AMPAR trafficking and turnover. Multiple functional molecules and signaling cascades are involved in the homeostatic up- or downregulation induced by prolonged activitydeprivation or overactivation, respectively. Elevated synaptic activation stimulates the expression of an immediate-early gene product, Arc, which, together with endophilin and dynamin, promotes AMPAR internalization. This process also requires the association of AMPARs with PICK1, and Nedd4-mediated receptor ubiquitination and proteasomal degradation (left panel). Under activity deprivation, varied signaling cascades are implicated in AMPAR trafficking and synaptic localization. Activation of TNFR1 by glia-derived TNF*α*, or PI3K pathway by BDNF/TrKBR and TNFR1 signaling, significantly enhances the postsynaptic AMPAR levels. *β*3 integrin signaling inhibits the activity of RAP1, which is known to enhance AMPAR degradation. Also, calcium influx through the inactivity-induced GluA2-lacking AMPARs (Cp-AMPAR) activates the CaMKII pathway, causing AMPAR phosphorylation and insertion. In addition, retinoic acid receptor *α* (RAR*α*) and FMRP mediate AMPAR local synthesis in postsynaptic domain during activity deprivation-induced upscaling (right panel).

Recent studies have shown that different functional molecules and signaling cascades are involved in the expression of homeostatic up- or downregulation of synaptic activity and AMPAR expression. For instance, TNF*α*, the PI3K-Akt pathway, integrin, GluA2-lacking AMPARs (Cp-AMPARs), and retinoic acid have been implicated in inactivity-induced homeostatic upscaling, whereas PICK1, a postsynaptic scaffolding protein, is involved in overexcitation-induced downscaling. Other molecules, such as CaMKs, Arc/Arg3.1, and certain cell adhesion molecules, have been implicated in both directions of homeostatic regulation. Given that AMPARs are the substrate for the expression of homeostatic plasticity, it is not surprising that most of these molecules and cascades are known to play an important role in AMPAR trafficking and synaptic accumulation. This paper summarizes recent findings on the signaling molecules and cascades that regulate AMPAR trafficking in homeostatic plasticity (Figure 3).

### 4.1. TNF*α*

Tumor necrosis factor-alpha (TNF*α*) is an inflammatory cytokine that is involved in inflammation, immune activation, cell death, and degradation [22, 23]. In addition to its important functions in immune responses, TNF*α* is also important in maintaining the neural network stability [24, 25]. TNF*α* has been found to mediate the global homeostatic upscaling of mEPSCs and postsynaptic AMPARs induced by prolonged TTX treatment [24]. When incubated with medium obtained from TTX-treated neural cell cultures, cultured hippocampal neurons globally scale up mEPSC amplitudes and postsynaptic AMPAR numbers. This effect can be abolished by applying exogenous high-affinity TNF*α* receptors to scavenge TNF*α* from the medium, indicating a critical role of free TNF*α* released to the extracellular environment to mediate synaptic upscaling during activity deprivation. To further support TNF*α* signaling in homeostatic plasticity, TTX-induced global upscaling in mEPSC is completely abolished in cultured hippocampal neurons or brain slices from TNF*α* knockout mice [24]. Interestingly, this abolished scaling can be rescued by coculture of TNF*α* knockout neurons with wild-type glial cells. However, although neurons produce TNF*α* by themselves, coculture of wild-type neurons with TNF*α* knockout glial cells still abolishes the upscaling in wild-type neurons [24]. Therefore, TNF*α* released from glia, but not neurons, plays a crucial role in the induction of global, slow homeostatic synaptic upregulation.

TNF*α* seems to mediate TTX-induced synaptic upscaling by regulating the trafficking and synaptic accumulation of AMPARs. Studies have shown that application of TNF*α* in cultured hippocampal neurons induces a rapid translocation of AMPARs (within 15 min) to the postsynaptic domain in a subunit-specific manner [26, 27]. After TNF*α* treatment, the delivery of AMPARs to postsynaptic surface is enhanced significantly, through downstream activation of PI3K pathway [28]. It also has been found that the effects of TNF*α* on AMPAR trafficking are mainly through TNFR1 but not TNFR2 [29]. Of note, the enhanced delivery of GluA1 subunit occurs faster than GluA2, leading to the generation of GluA2-lacking AMPARs. Calcium influx through these special type of AMPARs is believed to have an important role in the initiation of homeostatic response [30] (see Section 4.6 on Cp-AMPARs). In addition to enhancing AMPAR synaptic delivery, TNF*α* also decreases the trafficking of GABA_A_ receptors to the synapses [27]. Thus, TNF*α*-mediated synaptic homeostatic regulation of neuronal activity could be achieved via rebalancing excitation and inhibition.

### 4.2. CaMKs

Calcium (Ca^2+^) is one of the most important signaling molecules in the nervous system. In neurons, Ca^2+^ transients from ligand- and voltage-gated calcium channels and intracellular calcium stores activate a family of Ser/Thr protein kinases known as Ca^2+^/calmodulin-dependent protein kinases (CaMKs) to execute signaling functions [31, 32]. Among all CaMKs, CaMKII and CaMKIV are mostly known to be closely involved in synaptic transmission and different forms of synaptic plasticity by regulating receptor synthesis and trafficking [32, 33], through their ability to either directly phosphorylate AMPARs (CaMKII) or to regulate Ca^2+^-stimulated gene expression [34–36]. Although the important role of CaMKs in Hebbian synaptic plasticity has been extensively studied [32, 37], their involvement in homeostatic regulation has not been studied until recently. In a cultured cortical neuron, Ibata et al. inhibit single neuron firing by local perfusion of TTX on the cell body. They show that 4 hr selective inhibition of somatic activity induces a rapid, global upscaling in mEPSC amplitude and postsynaptic AMPARs in the inhibited neuron [16]. The quantity of surface AMPARs is imaged and measured by live imaging of EYFP-tagged GluA2 subunits expressed in dissociated rat cortical neurons. This homeostatic response results from a drop in somatic Ca^2+^ level and reduced CaMKIV activation. Coexpression of a dominant-negative form of CaMKIV (dnCaMKIV) with EYFP-GluA2 for 24 hrs mimics the TTX effect on mEPSCs [16]. In another study, Goold and Nicoll employ an optogenetic technique to activate individual neurons. Channelrhodopsin (ChR2) is expressed in hippocampal CA1 neurons in brain slices for 2-3 days, and a light train at 3 Hz is applied to stimulate the transfected neurons for 24 hrs. This lasting activation leads to a homeostatic depression of both AMPAR- and NMDAR-mediated currents, and a reduction in surface expression of AMPARs and NMDARs [17]. CaMKIV, activated by Ca^2+^ influx through the L-type voltage-gated calcium channels, plays a key role in the light activation-induced responses; expression of a dominant-negative CaMKIV blocks the homeostatic downregulation in AMPARs and NMDARs [17]. Together, these studies indicate an important role for CaMKIV in cell-autonomous bidirectional homeostatic plasticity.

In addition to CaMKIV, other CaMK members, like CaMKII, may also participate in homeostatic regulation. Thiagarajan et al. show that *α*- and *β*CaMKII are inversely regulated by neuronal activity and they also have opposite effects on mEPSCs [38]. In cultured hippocampal neurons, activity deprivation by 24-hour TTX treatment significantly decreases *α*CaMKII but increases *β*CaMKII expression. Conversely, chronic hyperactivity by 24-hour bicuculline treatment significantly increases *α*CaMKII and decreased *β*CaMKII levels. Overexpression of *α*CaMKII in hippocampal neurons significantly decreases, while *β*CaMKII increases, the amplitude of mEPSCs after 20–30 hrs [38]. Recently, another study shows that knockdown of *β*CaMKII in hippocampal neuron blocks NBQX-induced homeostatic increase in synaptic AMPAR expression whereas *β*CaMKII overexpression increases synaptic AMPAR levels [39]. These findings suggest an important role of CaMKII in the expression of homeostatic synaptic response.

### 4.3. BDNF

Brain-derived neurotrophic factor (BDNF) is broadly involved in many physiological processes in the developing or mature nervous system. BDNF is released from neurons in an activity- and calcium-dependent manner [40]. Once released, BDNF binds to the TrKB receptors to trigger a series of downstream signaling pathways [41]. Given its positive role in neuroprotection, BDNF is considered valuable in the management of several neurological diseases such as Huntington's disease, epilepsy, and Alzheimer's Disease [42].

The chronic presence of BDNF enhances synaptogenesis [41] and controls synaptic transmission and plasticity at both glutamatergic and GABAergic synapses [43]. In cultured cortical neurons derived from rat visual cortex, BDNF is first found to mediate homeostatic downregulation of mEPSCs [44]. Application of exogenous BDNF blocks activity deprivation-induced homeostatic upscaling, whereas BDNF depletion by high-affinity TrKB receptors mimics inactivity-induced upward homeostatic change in mEPSCs [44]. BDNF has been found to affect mEPSCs differentially in pyramidal neurons and interneurons [44]. In cultured rat visual cortical neurons, BDNF attenuates the TTX-induced homeostatic increase in mEPSC amplitudes at synapses formed onto pyramidal neurons, but enhances the TTX effect at synapses formed onto interneurons [44]. Intriguingly, in cultured hippocampal neurons, BDNF treatment enhances mEPSCs in excitatory synapses, likely due to enhanced AMPAR trafficking [45, 46], which is inconsistent with its role in homeostatic downregulation. Recently, several studies show that BDNF treatment enhances AMPAR trafficking to glutamatergic synapses, both *in vitro* and *in vivo* [47, 48]. In cultured hippocampal neurons, Caldeira et al. show that elevated AMPAR trafficking by BDNF is receptor subunit specific. Surface expression of GluA1 is preferentially increased in the first 30 min of BDNF incubation, leading to the formation of calcium-permeable, GluA1 homomeric AMPARs. At a later stage, BDNF incubation enhances the delivery of GluA2 and GluA3 subunits [47]. Enhanced AMPAR synaptic delivery requires the activation of TrKB receptors and the PI3K-Akt pathway [49], most likely through phosphorylation of the GluA1 C-terminal at S831 [47].

In addition to its role in AMPAR trafficking, BDNF has also been shown to function as a retrograde messenger released from the postsynaptic site to alter presynaptic activity in an activity-dependent manner [50, 51]. Inhibition of AMPAR activity by NBQX for 24 hrs or CNQX for 3 hrs homeostatically increases the frequency and amplitude of mEPSCs. The increase of mEPSC frequency is directly mediated by the retrograde signaling of BDNF. Removal of BDNF by adding high-affinity TrKB receptors or BDNF antibodies and pharmacologically blocking of the down-stream function of BDNF all lead to an abolishment of the enhanced presynaptic response without affecting postsynaptic activity. Consistently, application of BDNF itself induces similar presynaptic changes as produced by AMPAR inhibition [51].

### 4.4. PI3K-Akt Pathway

The phosphoinositide 3-kinase- (PI3K-) Akt pathway is wellknown for its involvement in AMPAR trafficking, synaptic plasticity, and memory consolidation both *in vivo* and *in vitro* [52, 53]. For instance, protein synthesis and AMPAR insertion in late-phase LTP requires PI3K pathway activation [53–55]. *In vivo*, activation of the PI3K pathway is also required for the maintenance of LTP in hippocampal CA1 neurons [53]. Consistent with its important role in LTP, PI3K is implicated in memory consolidation as well [52]. However, in contrast to abundant evidence about its role in Hebbian synaptic plasticity, involvement of PI3K signaling in homeostatic synaptic plasticity remains unclear. The PI3K cascade is potentially a good mediator in homeostatic regulation. First, the PI3K-Akt pathway is known to induce protein synthesis [55] and AMPAR membrane insertion [54, 56], processes known to occur in homeostatic AMPAR upregulation [19]. Second, both TNF*α* and BDNF, the two major factors that mediate homeostatic plasticity, activate the PI3K-Akt pathway and increase AMPAR expression at the postsynaptic surface [28, 41, 49]. Indeed, work by Hou et al. first revealed a requirement of PI3K in homeostatic response. In cultured hippocampal neurons, inhibition of presynaptic activity by overexpression of an inward-rectifier potassium channel Kir2.1 results in a significant increase in postsynaptic AMPARs. This homeostatic upregulation of AMPARs is abolished by application of a PI3K inhibitor wortmannin [20]. Consistent with this finding, a recent study also demonstrates the PI3K-Akt pathway as a mediator of global homeostatic plasticity. Presenilin 1 (PS1) is an integral component of *γ*-secretase that is closely linked to Alzheimer's disease [57, 58]. PS1 activates the PI3K-Akt pathway by promoting the formation of Akt-activating cadherin/PI3K complexes [59], Pratt et al. have found that the global homeostatic upscaling of mEPSCs induced by prolonged TTX treatment is impaired in cultured hippocampal neurons derived from PS1 knockout mice, suggesting a possible involvement of PI3K cascade [60]. In support of this possibility, overexpression of constitutively active Akt rescues impaired global synaptic scaling in PS1 knock-out neurons without affecting mEPSC amplitude in wild-type neurons [60]. Together these studies indicate the necessity of the PI3K-Akt pathway in homeostatic upregulation.

### 4.5. Cell Adhesion Molecules

Integrins and neuronal (N)-cadherin/*β*-catenin are cell adhesion molecules (CAMs) that are expressed at synapses and possess important functions in synapse formation, differentiation, and maturation, as well as synaptic plasticity [61, 62]. Malfunction of CAMs can produce severe problems in the nervous system including abnormal spine morphology, decreased synapses quantity, and impaired cognitive function [61, 63–66]. N-cadherin is a Ca^2+^-dependent homophilic adhesion protein that is present at both pre- and postsynaptic membranes [67]. It signals through Rho-family GTPases, via catenins, to control dendritic spine morphology and motility [68]. N-cadherin forms a complex with *β*-catenin in the synapse. The N-cadherin/*β*-catenin complex regulates the surface expression and intracellular trafficking of AMPARs by directly interacting with AMPARs [69–71]. The N-terminal domain of N-cadherin physically interacts with the GluA2 subunit of AMPARs at the extracellular space, promoting spine growth and synaptic transmission [71]. Nuriya and Huganir have shown that overexpression of wild-type N-cadherin in cultured hippocampal neurons can specifically increase the postsynaptic AMPAR expression [69]. By targeting AMPARs during endocytosis and exocytosis at the postsynaptic surface, the N-cadherin/*β*-catenin complexes also regulate the bidirectional homeostatic responses of neural activity. Okuda et al. show over-expression of a dominant-negative mutant of N-cadherin compromises the chronic up-scaling of quantal AMPAR responses induced by TTX treatment, whereas selective deletion of *β*-catenin in cultured hippocampal neurons eliminates the global homeostatic regulation [72, 73]. In addition to their roles in conventional postsynaptic homeostatic regulation, a recent study has demonstrated a role for N-cadherin/*β*-catenin in the compensatory adaptation of presynaptic release to chronic activity deprivation by TTX treatment. Vitureira et al. have found overexpression of dominant-negative N-cadherin (DN-NCad) in cultured hippocampal neurons significantly reduces the basal presynaptic release probability, but does not change the homeostatic upregulation of presynaptic release [73]. In contrast, ablation of *β*-catenin has no effect on basal presynaptic release but completely abolishes the homeostatic increase in presynaptic release induced by chronic activity deprivation by TTX treatment [73].

Integrins are heterodimeric transmembrane molecules of which many subunits are expressed in the central nervous system. Previous studies have revealed multiple roles for integrins in synaptogenesis, synaptic transmission, and plasticity, as well as memory formation [65, 74]. *β*3 integrin regulates synaptic strength by modulating the surface expression of AMPARs in a subunit-specific manner [75]. Over-expression of *β*3 integrin in dissociated hippocampal neurons enhances AMPAR surface expression via suppression of RAP1 [75], a pathway known to negatively control AMPAR trafficking during synaptic plasticity by enhancing endocytosis of GluA2 subunits [76]. Because TNF*α*, an important molecule in homeostatic plasticity, causes an elevated cell-surface expression of *β*3 integrin, it suggests a possible involvement of *β*3 integrin in homeostatic response by regulating AMPAR trafficking. Consistent with this hypothesis, a recent study shows that 24 hr TTX or bicuculline treatment causes a corresponding increase or decrease of surface *β*3 integrin without affecting *β*1 integrin [75]. Prolonged TTX treatment enhances the surface *β*3 integrin to a level similar to that by TNF*α* treatment. More directly, overexpression of dominant-negative *β*3 integrin, CT*β*3, completely eliminates the TTX-induced global homeostatic up-scaling of mEPSCs and synaptic AMPAR expression [75].

### 4.6. GluA2-Lacking, Calcium-Permeable AMPARs (Cp-AMPARs)

Under physiological conditions most AMPARs contain at least one GluA2 subunit and allow only sodium influx to depolarize membrane potential during synaptic activation. When AMPARs are composed without GluA2 subunits, the receptor channel will permeate calcium in addition to sodium. Due to a channel blockade by intracellular polyamine at positive membrane potential, currents from Cp-AMPARs show a signature feature of inward rectification in the current-voltage relationship. It has been found that the expression of GluA2-lacking receptors is regulated by development, synaptic activity, or pathological challenges such as ischemia or amyotrophic lateral sclerosis (ALS) [77]. In the early postnatal ages, cortical pyramidal neurons have higher rectification in AMPAR-mediated synaptic currents, which diminishes in more mature animals, indicating a developmental switch in AMPAR composition and calcium permeability [78]. By providing an unconventional source of calcium other than NMDAR or calcium channels, GluA2-lacking AMPARs may play an important role in synaptic plasticity. Indeed, in the expression of hippocampal LTP, GluA2-lacking AMPARs are first incorporated into the synapse, which will then be replaced with GluA2-containing receptors [79]. In cerebellar stellate cells which normally contain GluA2-lacking AMPARs, high-frequency presynaptic activity induces a calcium-dependent increase in synaptic insertion of GluA2-containing AMPARs [80]. These findings strongly indicate the presence of a self-regulating mechanism by which Cp-AMPAR-mediated calcium flux triggers recruitment of normal GluA2-containing AMPARs to synapses.

Activity deprivation has been shown to induce inward rectification in the current-voltage relationship of AMPAR-mediated current, suggesting the formation of GluA2-lacking, calcium-permeable AMPARs [19, 81–83]. Consistently, following treatment paradigms for homeostatic plasticity, AMPAR-mediated currents become sensitive to Cp-AMPAR-selective antagonists philanthotoxin-433 (PhTx) or Naspm [19, 20, 81–83]. Interestingly, multiple signaling molecules that are involved in homeostatic synaptic plasticity including TNF*α*, retinoic acid, Arc/Arg3.1, and *β*3 integrin are capable of causing imbalanced GluA1 and GluA2 regulation and Cp-AMPAR expression. The homeostatic factor TNF*α* is known to cause rapid membrane insertion of GluA2-lacking AMPARs [28, 84]. In retinoic acid-mediated synaptic scaling, the increase in AMPAR surface expression is GluA1 specific, and the homeostatic response in mEPSCs is abolished by suppression of Cp-AMPARs [82]. An unbalanced regulation in AMPAR subunits is also observed in Arc/Arg3.1-mediated homeostatic regulation. Knockout of Arc/Arg3.1 results in a typical synaptic scaling of AMPAR-mediated mEPSCs. Interestingly, Arc/Arg3.1 knockout neurons reveal a significant increase in GluA1 surface expression, whereas surface GluA2 shows no change [85], implicating membrane addition of GluA2-lacking AMPARs. In addition, disruption of *β*3 integrin induces internalization of GluA2, but not GluA1 subunits, resulting in GluA2-lacking AMPARs at the cell surface. In our own study, we find that homeostatic regulation by single synaptic suppression is abolished by the application of PhTx, indicating the requirement of Cp-AMPAR signaling [20]. Interestingly, the blockade of homeostatic plasticity is observed only when PhTx is applied at the early stage of activity deprivation [20], indicating that Cp-AMPARs are needed for the initiation, but not maintenance, of homeostatic synaptic regulation.

### 4.7. Arc/Arg3.1

Arc/Arg3.1 is an immediate-early gene product whose abundance at synapses is strictly coupled with neural activity level. Strong synaptic activation will dramatically enhance the expression of Arc/Arg3.1 in dendrites and spines while synaptic suppression decreases its expression [86, 87]. Arc/Arg3.1 is broadly involved in different forms of synaptic plasticity including both Hebbian plasticity and homeostatic regulation [88]. Rial Verde et al. find that Arc/Arg3.1 controls synaptic transmission strength by negatively regulating the surface expression of AMPAR expression at the post-synaptic surface. Over-expression of Arc/Arg3.1 in hippocampal neurons promotes the endocytosis of GluA2/3 containing AMPARs as well as the reduction of AMPAR-mediated synaptic current amplitude. Importantly, knockdown of Arc/Arg3.1 by siRNA abolishes this effect [87]. In Arc/Arg3.1 knockout mice, AMPARs show markedly reduced endocytosis and enhanced steady-state surface expression [85, 89]. Shepherd et al. also find that Arc/Arg3.1 is involved in bidirectional homeostatic regulation of neural activity via regulating AMPAR internalization and endocytosis [85]. In primary neuronal culture, synaptic expression of Arc/Arg3.1 is enhanced after chronic activity deprivation, while over-expression of Arc/Arg3.1 blocks the global synaptic up-scaling of mEPSCs and AMPARs induced by chronic activity deprivation. Conversely, in cultured hippocampal neurons from Arc/Arg3.1 knockout (KO) mice, either global up- or down-homeostatic scaling induced by prolonged TTX or bicuculline treatment is impaired [85]. Together these studies demonstrate strong evidence about the involvement of Arc/Arg3.1 in the bi-directional homeostatic regulation of neural activity through regulation of the trafficking of AMPARs. It has also been shown that Arc/Arg3.1 directly interacts with endophilin and dynamin to form post-synaptic endosomes which facilitate the endocytosis of AMPARs [89]. In addition to global regulation, Arc/Arg3.1 also mediates local homeostatic plasticity at individual synapses. Using Kir2.1 paradigm, Béïque et al. show that homeostatic upregulation of mEPSCs is abolished in cultured cortical pyramidal neurons derived from Arc knock-out mice [90].

### 4.8. Retinoic Acid and FMRP

Retinoic acid (RA), also known as Vitamin A, is best known for its role in regulating the development of the nervous system, including neurogenesis and neuronal differentiation [91, 92]. In a recent study, Aoto et al. show that 24 hr TTX + APV treatment, a homeostatic paradigm that induces global synaptic upscaling of mEPSCs and AMPARs [19], significantly enhances the synthesis of RA in both cultured hippocampal neurons and brain slices [82]. Application of RA rapidly increases the strength of synaptic transmission mainly through an increase in surface expression of AMPARs [82]. Effects of RA are translation, but not transcription dependent [93], and are occluded by TTX + APV treatment, indicating an involvement of RA signaling in homeostatic plasticity. In addition, AMPAR upregulation by APV + TTX treatment is subunit specific, with a preferential increase in GluA1 over GluA2, leading to the production of GluA2-lacking, calcium-permeable AMPARs [19]. A recent study from the same group indicates a role of the fragile-X mental retardation protein (FMRP) in RA-induced GluA1 local translation [94]. FMRP is a dendritic RNA-binding protein encoded by the Fmr1 gene that is involved in the downregulation of local mRNA translation and protein synthesis [95, 96]. In Fmr1 knockout mice, both TTX + APV-induced AMPAR homeostatic upregulation and RA-induced local AMPAR synthesis are impaired. Over-expression of WT-FMRP but not mutant FMRP in Fmr1 knockout neurons restores the impaired homeostatic upscaling of mEPSCs and AMPARs [94]. Therefore, via the effect of FMRP, homeostatic regulation may be implicated in the neurodysfunction in fragile X syndrome.

### 4.9. PICK1

Protein interacting with C-kinase 1 (PICK1) is a PDZ domain-containing protein that directly interacts with the GluA2 subunit of AMPARs [97, 98]. PICK1 also directly interacts with the AMPAR adaptor protein, ABP/GRIP to regulate AMPAR trafficking [99]. The importance of PICK1 in Hebbian synaptic plasticity, especially in LTD, has been well documented both *in vivo* and *in vitro* [100, 101]. PICK1 influences synaptic plasticity by stimulating AMPAR internalization [102, 103]. For instance, in PICK1 knockout mice, NMDA-induced LTD is abolished in hippocampal neurons due to disrupted internalization, recycling, and retention of GluA2-containing AMPARs [100, 101]. By interacting with the GluA2 subunit, PICK1 plays a key role in the plasticity involving the calcium-permeable, GluA2-lacking AMPARs [104]. In a specific type of LTP induced by cocaine exposure at the glutamatergic synapses of dopaminergic neurons in the ventral tegmental area, PICK1 directly mediates the switch of GluA2-containing to GluA2-lacking AMPARs [105]. Since calcium-permeable AMPARs serve as an important signal in homeostatic scaling [30], these findings imply a regulatory role for PICK1 in homeostatic regulation. Indeed, a recent study using cultured cortical neurons showed that PICK1 specifically mediates the TTX-induced global up-scaling of mEPSCs and synaptic AMPARs, without affecting bicuculline-induced downward homeostatic regulation [106]. In cultured cortical pyramidal neurons derived from PICK1 knockout mice, while the number of synaptic AMPARs are increased, TTX-induced mEPSC up-scaling is occluded due to altered AMPAR subunit composition and aberrant receptor trafficking [106].

### 4.10. Ubiquitin-Proteasome System

The ubiquitin-proteasome system (UPS) is a crucial proteolytic mechanism. UPS uses a small protein of 76 amino acids, namely, ubiquitin, to mark proteins destined for degradation. Following ubiquitination, a polyubiquitin chain is attached to the lysine residues of the target protein so that it can be recognized by the degradation machinery proteasome [107]. UPS components are widely distributed in a neuron from the soma to dendrites and synapses [107, 108]. It regulates many important synaptic functions including synapse development, maturation, and synaptic plasticity [109]. Given the importance of proper protein turnover in cells, UPS dysfunction is implicated in the pathogenesis of many neurodegenerative diseases [110].

UPS function is closely related to the neural activity levels [108, 111] to control the post-synaptic proteins composition including PSD-95 [112, 113] and AMPAR-associating protein GRIP [114]. AMPARs are directly subjected to ubiquitination, leading to their internalization and degradation [115–117]. The degradation of NMDARs is also regulated by the UPS [118]. Several recent studies have shown the UPS activity is also involved in the regulation of both global and local homeostatic plasticity. Jakawich et al. show that application of a proteasome inhibitor, lactacystin, for 24 hrs in cultured hippocampal neurons causes a global up-scaling of mEPSCs and AMPAR expression, which mimics and occludes the TTX-induced homeostatic synaptic response [119]. In addition to the pharmacological study, expression of a mutant ubiquitin which inhibits protein ubiquitination by blocking ubiquitin chain elongation produces similar effects as prolonged lactacystin treatment [119]. In another recent study, Hou et al. utilize light-controlled glutamate receptor (LiGluR) to selectively activate individual synapses in cultured hippocampal neurons. Single synaptic activation leads to homeostatic downregulation of postsynaptic AMPAR abundance as a consequence of enhanced AMPAR internalization and degradation [18]. This activity-dependent homeostatic AMPAR alteration is accompanied by a recruitment of polyubiquitinated proteins and AMPAR E3 ligase Nedd4 [115, 116, 120] and is blocked by the application of proteasome inhibitors, strongly indicating a key role of the UPS in hyperactivity-induced homeostatic plasticity [18].

## 5. Conclusion

Homeostatic synaptic regulation is one of the fundamental forms of plasticity serving to maintain the functional stability of the nervous system from a single synapse to an entire neural network. At a cellular level, homeostatic response will keep the firing rate within a physiological range, and at individual synapses, it prevents synaptic activity from running away to the extremes. Although a change in AMPAR trafficking and synaptic accumulation has been considered the primary mechanism, homeostatic regulation of neuronal activity can also be expressed via regulating other cellular components such as presynaptic transmitter release, cell intrinsic excitability, synaptic connectivity, and the relative balance between excitation and inhibition. Potentially, dysfunction of homeostatic regulation will lead to a shift in basal neuronal and synaptic activity, and altered sensitivity to stimuli. Consequently, super up-scaling could cause epileptic activity and excitotoxic cell death, whereas abnormal downscaling would result in neural suppression and impaired cognitive function. Therefore, homeostatic plasticity may play a crucial role in the pathogenesis of multiple neurological disorders including neurodegenerative diseases, which, we expect, will continue to be a topic of investigation for the foreseeable future.
